# *European Journal of Hybrid Imaging*: the journey continues

**DOI:** 10.1186/s41824-018-0049-8

**Published:** 2019-01-11

**Authors:** Arturo Chiti, Francesco Giammarile

**Affiliations:** Department of Biomedical Sciences, Humanitas University, Nuclear Medicine Unit – Humanitas Clinical and Research Center – IRCCS, Via Rita Levi Montalcini 4 – 20090 Pieve Emanuele, Milano, Italy

The *European Journal of Hybrid Imaging* (*EJHI*) opened to contributions in February 2017 and was launched in October of the same year, as a platform to publish papers focused on the use of hybrid and multimodality imaging in clinical practice, clinical trials, and pre-clinical research. *EJHI* was born as the last member of the EJNMMI family of journals and is now well integrated into the scientific workflow of the family.

As an Open Access journal, *EJHI* can ensure the rapid and widespread distribution of published articles. The open access model offers a fast and reliable way to disseminate knowledge, and publication can be supported with several funding schemes when authors have no resources available to them. In this respect, the European Association of Nuclear Medicine (EANM) has supported the publication of relevant papers with a sponsorship program. The EANM program helped authors who would otherwise lack Open Access funding publish in *EJHI*, and the resulting research has contributed greatly to both to the field and the success of the journal. This program will be continued next year, in order to facilitate the successful positioning of the journal in the scientific community.

In less than 2 years of life, the *EJHI* has received contributions not only from the nuclear medicine community but also from authors in other disciplines that involve hybrid imaging.

As of September 2018, the *EJHI* received 49 manuscripts, of which 37 were accepted and 3 are currently under revision. In 2017 only, the articles published in *EJHI* were downloaded 587 times, with a usage factor of 122.

A fast and effective peer review process is practiced at the journal, in line with the characteristics of the EJNMMI family of journals, and this ensures the rapid appraisal of manuscripts.

When taken together, we have all the arguments to say that the *European Journal of Hybrid Imaging* has a brief, but very successful, history.

To continue this trend, Springer Nature and EANM, in agreement with Arturo Chiti, have asked Francesco Giammarile to become the Editor-in-Chief of the *European Journal of Hybrid Imaging*, as of January 1, 2019. At the same time, Arturo Chiti will become the Editor-in-Chief of the *European Journal of Nuclear Medicine and Molecular Imaging*, continuing his efforts in the EANM family of journals.

Prof. Giammarile will ensure a smooth transition and the continued growth of the *EJHI*, driving the journal towards an established position within the scientific journals in its field.

Arturo Chiti, Humanitas University and Humanitas Clinical and Research Center, Milan, Italy.

Francesco Giammarile, International Atomic Energy Agency, Vienna, Austria.

